# How often do mosquitoes bite humans in southern England? A standardised summer trial at four sites reveals spatial, temporal and site-related variation in biting rates

**DOI:** 10.1186/s13071-017-2360-9

**Published:** 2017-09-15

**Authors:** Victor A. Brugman, Marion E. England, Joanne Stoner, Laura Tugwell, Lara E. Harrup, Anthony J. Wilson, Jolyon M. Medlock, James G. Logan, Anthony R. Fooks, Peter P.C. Mertens, Nicholas Johnson, Simon Carpenter

**Affiliations:** 1 The Pirbright Institute, Ash Road, Woking, Surrey, UK; 2 London School of Hygiene and Tropical Medicine, Keppel Street, London, UK; 3 Public Health England, Porton Down, Salisbury, UK; 4 Health Protection Research Unit in Emerging Infections & Zoonoses, Porton Down, Salisbury, UK; 5 Animal and Plant Health Agency, New Haw, Addlestone, Surrey, UK; 6 Department of Clinical Infection, Microbiology and Immunology, University of Liverpool, Liverpool, UK; 7 The University of Nottingham, Sutton Bonington, Leicestershire, UK; 8 Faculty of Health and Medical Sciences, University of Surrey, Guildford, Surrey, UK

**Keywords:** Mosquito, Biting rate, Human landing catch, *Culex*, *Coquillettidia*, Blood meal

## Abstract

**Background:**

This field-based study examined the abundance and species complement of mosquitoes (Diptera: Culicidae) attracted to humans at four sites in the United Kingdom (UK). The study used a systematic approach to directly measure feeding by mosquitoes on humans at multiple sites and using multiple volunteers. Quantifying how frequently humans are bitten in the field by mosquitoes is a fundamental parameter in assessing arthropod-borne virus transmission.

**Methods:**

Human landing catches were conducted using a standardised protocol by multiple volunteers at four rural sites between July and August 2013. Collections commenced two hours prior to sunset and lasted for a total of four hours. To reduce bias occurring due to collection point or to the individual attractiveness of the volunteer to mosquitoes, each collection was divided into eight collection periods, with volunteers rotated by randomised Latin square design between four sampling points per site. While the aim was to collect mosquitoes prior to feeding, the source of blood meals from any engorged specimens was also identified by DNA barcoding.

**Results:**

Three of the four sites yielded human-biting mosquito populations for a total of 915 mosquitoes of fifteen species/species groups. Mosquito species composition and biting rates differed significantly between sites, with individual volunteers collecting between 0 and 89 mosquitoes (over 200 per hour) of up to six species per collection period. *Coquillettidia richiardii* (Ficalbi, 1889) was responsible for the highest recorded biting rates at any one site, reaching 161 bites per hour, whilst maximum biting rates of 55 bites per hour were recorded for *Culex modestus* (Ficalbi, 1889). Human-biting by *Culex pipiens* (L., 1758) form *pipiens* was also observed at two sites, but at much lower rates when compared to other species.

**Conclusions:**

Several mosquito species are responsible for human nuisance biting pressure in southern England, although human exposure to biting may be largely limited to evening outdoor activities. This study indicates *Cx. modestus* can be a major human-biting species in the UK whilst *Cx. pipiens* f. *pipiens* may show greater opportunistic human-biting than indicated by earlier studies.

**Electronic supplementary material:**

The online version of this article (10.1186/s13071-017-2360-9) contains supplementary material, which is available to authorized users.

## Background

Direct studies of mosquitoes biting human populations are important in understanding the impact of biting nuisance and transmission rates for zoonotic and anthroponotic mosquito-borne pathogens globally [1]. In the UK, nuisance biting of humans by mosquitoes can be a significant and often overlooked problem in both rural and urban environments [2–4]. Furthermore, the emergence and re-emergence of mosquito-borne pathogens are considered significant threats to the UK [5, 6]. To date, at least 23 native mosquito species have been reported to bite humans in the UK (Table 1). This list includes several species that are proven or implicated vectors of important zoonotic and medical pathogens in Europe, including *Plasmodium vivax* [*Anopheles maculipennis* (*sensu lato*) (*s.l.*), comprised of three species: *An. atroparvus* (van Thiel, 1927), *An. messeae* (Falleroni, 1926) and *An. daciae* (Linton, Nicolescu & Harbach, 2004)], *P. falciparum* [*Anopheles plumbeus* (Stephens, 1828)] and several arthropod-borne viruses (arboviruses) [notably *Culex* and *Aedes* spp.]. No local human infection with mosquito-borne arboviruses has been reported in the UK for 150 years but indigenous mosquito species are competent vectors for some viruses [7–11], and the potential for emergence remains [5, 12].

**Table 1:** Reported human-biting behaviour of mosquitoes within literature in the United Kingdom: published literature concerning human-biting mosquitoes, categorised according to biting nuisance reports, blood meal analysis studies and human-baited collections

Mosquito species	Published evidence for human-biting behaviour in the UK		
	Biting nuisance reports	Blood meal analysis	Human-baited collections
*Aedes* (*Aedes*) *cinereus* (Meigen, 1818)/*Aedes* (*Aedes*) *geminus* (Peus, 1970)^a^	Yes [75]	Yes [19, 20]	Yes [19, 20, 68]
*Aedes* (*Aedimorphus*) *vexans* (Meigen, 1830)	Yes [75]	–	–
*Anopheles* (*Anopheles*) *algeriensis* (Theobald, 1903)	Yes [76]	–	–
*Anopheles* (*Anopheles*) *claviger* (Meigen, 1804)	Yes [77, 78]	–	Yes [20]
*Anopheles* (*Anopheles*) *maculipennis* (*s.l.*)^b^	Yes [3, 75]	Yes [79]^d^	–
*Anopheles* (*Anopheles*) *plumbeus* (Stephens, 1828)	Yes [75]	Yes [19, 20]	Yes [19, 20, 68]
*Coquillettidia* (*Coquillettidia*) *richiardii* (Ficalbi, 1889)	Yes [3]	Yes [19, 20, 61]	Yes [19, 20, 68]
*Culex* (*Barraudius*) *modestus* (Ficalbi, 1889)	–	Yes [61]	Yes [60]^e^
*Culex* (*Culex*) *pipiens* (L., 1758)^c^	Yes [3, 77, 78]	Yes [19, 20]	–
*Culiseta* (*Culiseta*) *annulata* (Schrank, 1776)	Yes [3, 75, 77, 78]	Yes [19, 20]	Yes [19, 20]
*Culiseta* (*Culicella*) *litorea* (Shute, 1928)	–	Yes [19, 20, 80]	–
*Culiseta* (*Culicella*) *morsitans* (Theobald, 1901)	–	Yes [19, 20, 80]	–
*Culiseta* (*Culiseta*) *subochrea* (Edwards, 1921)	Yes [3, 75]	–	–
*Aedes* (*Dahliana*) *geniculatus* (Olivier, 1791)	Yes [75]	Yes [20]	Yes [19, 20, 68, 81]
*Aedes* (*Ochlerotatus*) *annulipes* (Meigen, 1830)	Yes [3]	–	Yes [19]
*Aedes* (*Ochlerotatus*) *cantans* (Meigen, 1818)	Yes [3, 77, 78]	Yes [19, 20, 61, 82]	Yes [19, 20, 68, 82, 83]
*Aedes* (*Ochlerotatus*) *caspius* (Pallas, 1771)	Yes [75, 77, 78]	–	Yes [19]
*Aedes* (*Ochlerotatus*) *detritus* (Haliday, 1833)	Yes [3, 75, 77, 78]	Yes [19, 20, 61]	Yes [19, 20, 68]
*Aedes* (*Ochlerotatus*) *dorsalis* (Meigen, 1830)	Yes [75]	Yes	–
*Aedes* (*Ochlerotatus*) *flavescens* (Müller, 1764)	Yes [75]	–	–
*Aedes* (*Ochlerotatus*) *punctor* (Kirby, 1837)	Yes [3, 75, 84]	Yes [19, 20]	Yes [19, 20, 68]
*Aedes* (*Ochlerotatus*) *rusticus* (Rossi, 1790)	Yes [77, 78]	–	Yes [19, 20, 68]

^a^These species were only recently separated in [39] and therefore are considered together

^b^Studies that did not delineate *An. maculipennis* (*s.l.*) to species level

^c^Ecoforms of *Culex pipiens* (L., 1758) not separated

^d^This study found evidence of human-biting in all three members of *An. maculipennis* (*s.l.*)

^e^Not a host-baited study per se, but an incidental collection of one specimen biting the collector

The number of mosquitoes biting a host is a key parameter in epidemiological models of vector-borne pathogen transmission, and reduction of this rate is an important target for disease control and prevention initiatives [13, 14]. Temporal and spatial variation in mosquito biting rates within an environment can significantly impact the risk of human infection with a mosquito-borne pathogen [15]. Although recent evidence suggests that some artificial trapping approaches are able to sample certain mosquito species at a comparable rate to that of human-baited collections [16–18], the standardised human landing catch remains a valuable method for assessing true biting rates on human hosts. The use of human landing catch techniques is particularly useful in areas where pathogen transmission is not considered to occur, as experiments can proceed without exposing personnel to an increased risk of infection [1].

Despite the importance of field data on human-biting mosquito populations, information on mosquitoes biting humans in the UK is largely based on studies conducted over 50 years ago and from a limited number of geographical locations [19, 20]. The studies by Service [19, 20] were also conducted by a lone worker and thus may have led to bias in frequency of landing and biting [21, 22]. In addition, the confounding influence of morphologically cryptic species, such as *Aedes nigrinus* (Eckstein, 1818) and *Aedes sticticus* (Meigen, 1838), may have significantly influenced the findings of early studies which were unable to harness the recently developed molecular methods for delineating these species [23]. The Pipiens complex, in particular, is a morphologically cryptic species group that has been reported to exhibit significant variation in bionomics and human-biting rates. Human-biting populations of *Culex pipiens* form *pipiens* have been detected in Portugal [24], despite this species being considered primarily ornithophilic [25]. Moreover, *Cx. pipiens* pipiens/molestus hybrid forms will feed on mammals [26–28], and may also act as bridge vectors of viruses to humans if they exhibit a more generalist feeding behaviour [29].

At present, active UK surveillance activities for adult mosquitoes are primarily based on trapping using Mosquito Magnet® (Woodstream Corporation, Lititz, Pennsylvania, USA) and BG Sentinel traps (Biogents, Regensburg, Germany) [30, 31]. Specimens are collected over extended (> 1 day) time periods, and therefore it is not possible to use these data to determine the biting rate of mosquitoes on humans at that location. Addressing this knowledge gap with field-based studies is vital to increase our understanding of current mosquito biting behaviour, and will allow future comparison with behavioural changes that could result from climate change [12], anthropogenic changes to the environment [32] or the establishment of an exotic species, such as *Aedes albopictus* (Skuse, 1895), in the UK [33].

This study therefore aimed to determine the human-biting rate of mosquito species assemblages present on four sites in the south of England during peak evening mosquito activity periods [19, 20] and during the summer months when mosquitoes are most abundant [34]. Mosquitoes were collected by human landing catch using multiple collectors to account for individual variations in attractiveness to biting, and meteorological data were collected to account for environmental variation. The sites chosen were biased towards livestock farms and nature reserves, which provide a setting in which humans, livestock and wildlife interact daily. Farm-related activities necessitate human presence outdoors, and may last for long periods of time which could overlap with periods of peak mosquito activity during the summer months.

## Methods

### Study sites

**Fig. 1:**
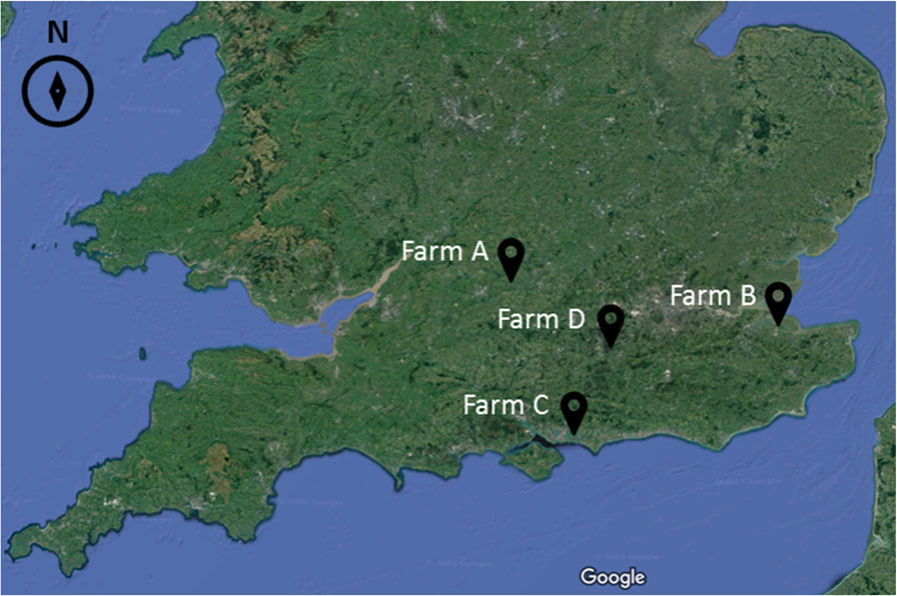
Location of study farms in southern England. Map: Google

The study was conducted on four sites (denoted A, B, C and D), located in Oxfordshire, Kent, Hampshire and Surrey, respectively (see Fig. 1 and Additional file 1: Table S1 for further details). These were selected based on their location in the south of England, the presence of livestock on site and the presence of mosquito larvae in either natural or artificial habitats in preliminary site visits conducted between June and September 2012 (data not shown). Within each site, four sampling points were chosen at which to conduct human landing catches. Sampling points were situated a minimum of 50 m apart, located in areas frequented by humans, in logistically feasible positions and in locations that presented minimal chances of interference with farm activities. To facilitate future characterisation and comparison between sites and studies, sampling points at sites B, C, and D were documented in the form of a Google Photosphere, a 360° photograph, captured using a Google Nexus 5 mobile phone. These files (Additional files 2, 3, 4, 5, 6, 7, 8, 9, 10, 11, 12 and 13) can be uploaded and viewed online at http://photosphereviewer.net/.

### Human landing catch protocol

**Fig. 2:**
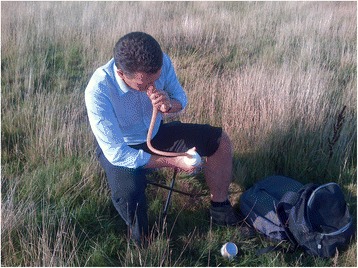
The human landing catch. A volunteer collector demonstrating a human landing catch at site C

The study took place between July and August 2013 over a total of 24 collection evenings, six at each site. Four collectors from a pool of thirteen were assigned to each evening, and asked to refrain from applying scented products (soaps, deodorants) on their collection days. Each night of collection ran for four hours starting two hours prior to sunset. The four-hour period was split into eight collection periods of 25 min each at the four sampling points, with five minutes available after each period for movement of the volunteer to the next sampling point. Collectors were assigned to a sampling point for each collection period (1–8) by two sequential Latin square randomisations using a script in R v.3.2.0 [35]. Landing catches were conducted while sitting on a stool and a manual aspirator (John W. Hock, Gainsville, Florida, USA) was used to collect any mosquitoes alighting on one exposed lower leg (Fig. 2). Collected mosquitoes were placed in a 64 mm diameter cardboard pill pot (Watkins and Doncaster, Leominster, UK). Only specimens that landed on the leg were aspirated. A red-light head torch (Petzl, Crolles, France) was used by collectors when natural light intensity was insufficient to carry out collections. At the end of each evening, mosquitoes were transported to the laboratory in a cooler containing dry ice and stored at −20 °C prior to further analysis.

### Collection of meteorological data

Trials were limited to evenings of average forecasted wind speeds below 3 m/s and minimal rainfall (< 1 mm) as forecasted by www.xcweather.co.uk, following criteria used previously as optimal for mosquito activity [19]. Meteorological data were collected at one-minute intervals from each site using an automatic weather station with data logger model CR800 (Campbell Scientific, Loughborough, UK). Variables collected were air temperature (°C), relative humidity (%), wind speed (m/s), solar intensity (kJ/m^2^) and rainfall (mm), with data for each of these variables summarised prior to analysis to a mean value per hour. Due to unexpected failure of the weather station at site B, temperature, wind speed and rainfall data at hourly intervals were obtained from the nearest Met Office weather station located approximately 20 km away at Shoeburyness, Essex, UK. The temperature/relative humidity probe at site D failed shortly before collections commenced and therefore temperature data were collected using a TinyTag Plus2 datalogger (Gemini Data Loggers Ltd., West Sussex, UK) hung at the same height as the standard probe.

### Identification of mosquitoes and blood meals

Mosquitoes were initially identified based on morphological characteristics using published keys [36, 37], according to the nomenclature of Wilkerson et al. [38]. Specimens difficult to separate morphologically and which display a broadly similar ecology [5, 36, 37, 39] were identified morphologically to species group only. Mosquitoes morphologically identified as *An. maculipennis* (*s.l.*) or *Cx. pipiens*/*Culex torrentium* (Martini, 1925) were subjected to molecular species delineation. DNA was extracted from the abdomens of the mosquitoes to facilitate both species delineation and blood meal analysis using previously established protocols [40, 41]. Specimens of *An. maculipennis* (*s.l.*) were identified to species-level by amplification of the 435-base pair (bp) region of the internal transcribed spacer 2 gene (ITS2) using primers 5.8S and 28S [42] in a polymerase chain reaction (PCR) assay, as described previously [40]. Sequences were assigned to a particular mosquito species when agreement was ≥ 98% to published sequences in GenBank, using the standard nucleotide BLAST tool [43]. To delineate *Cx. pipiens*/*Cx. torrentium*, specimens were subjected to two sequential duplex end-point PCR assays as described elsewhere [44]. To summarise, the first PCR assay separated *Cx. pipiens*/*Cx. torrentium* following the protocols of [44] and used the ACEtorr and ACEpip forward primers with the B1246s reverse primer, targeting the nuclear acetylcholinesterase-2 (*ace-2*) gene [45]. Specimens identified as *Cx. pipiens* were then subjected to a second duplex PCR assay to delineate between *Cx. pipiens* form *pipiens* and *Cx. pipiens* f. *molestus*. This PCR assay utilised the forward primer CQ11F and reverse primers molCQ11R and pipCQ11R targeting the CQ11 microsatellite locus [46, 47].

Blood meal host was determined using a six-primer cocktail (VF1_t1 + VF1d_t1 + VF1i_t1/VR1_t1 + VR1d_t1 + VR1i_t1) in an end-point PCR assay, targeting a 685 bp sequence of the mitochondrial cytochrome *c* oxidase subunit 1 (*cox*1) gene [48], following previously developed protocols [41]. Samples producing positive PCR results were sequenced uni-directionally with M13 primers [48] at 1 pmol/μl using the ABI PRISM® BigDye® Terminator v3.1 Cycle Sequencing Kit (Applied Biosystems, Warrington, UK), and sequences were assigned to a particular host when agreement was ≥ 98% to published sequences in GenBank using the standard nucleotide BLAST tool [43].

### Data analysis

Comparisons between sites and the effect of meteorological variables on the overall biting rate were assessed using generalized linear mixed models (GLMMs) in package ‘glmmADMB’ [49, 50] in R v.3.2.0 [35]. *Site* was included as a fixed effect, with *sampling point* within each site included as a random factor nested within site. The *collector* was also included as a random factor. *Time to sunset* (transformed to a squared factor), *temperature* and *wind speed* were included as covariates with *rainfall* fitted as a fixed presence/absence factor. Models were fitted by maximum likelihood with the Laplace approximation, and model fit assessed by comparison of Akaike information criterion (AIC) [51] in R, with lower values indicating a better model fit. The final model was obtained by step-wise deletion of non-significant factors and variables until removal caused an increase in AIC value of greater than two units.

## Results

### Human-biting rates and species composition

A total of 915 mosquitoes of fourteen species, or morphologically indistinguishable species groups, were collected in the study (Table 2). The greatest number of specimens was collected from site B (*n* = 802), followed by site C (*n* = 72) and site D (*n* = 41). No mosquitoes were collected at site A throughout the study and therefore this farm was excluded from further analysis. Mean human-biting rates per site, including all mosquito species (mosquitoes/person/25 min/night), were 4.18 (range 0–89 mosquitoes) at site B, 0.38 (0–9) at site C and 0.21 (0–6) at site D. The highest mean biting rates per 25 min collection period were for *Coquillettidia richiardii* (Ficalbi, 1889) (2.59, range 0–67 mosquitoes), *An. maculipennis* (*s.l.*) (0.28, 0–29) and *Cx. modestus* (Ficalbi, 1889) (1.04, 0–23). Extrapolated hourly biting rates (biting rate ÷ 25 × 60) for *Cq. richiardii*, *An. maculipennis* (*s.l.*) and *Cx. modestus* were 6.2 (range 0–161), 0.67 (0–70) and 2.5 (0–55), respectively. Of these species, only *Cq. richiardii* was collected from three sites (B, C and D) with *Cx. modestus* collected from site B only.

**Table 2:** Mosquitoes collected over the course of the study at the sites: the number (*n*), percentage composition (%), mean and range of biting rates at each farm. Mean values represent the number of bites per person, per 25 min, on an average night. The range represents the minimum and maximum number of specimens collected by one collector in any one 25 min period. Farm A is excluded as no mosquitoes were collected there during the study

Species	Site B		Site C		Site D		Total
	*n* (%)	Mean biting rate (range)	*n* (%)	Mean biting rate (range)	*n* (%)	Mean biting rate (range)	
*Aedes cinereus*/*geminus*	0 (0)	–	0 (0)	–	2 (4.9)	0.01 (0–1)	2
*Anopheles claviger*	3 (0.4)	0.02 (0–1)	1 (1.4)	0.01 (0–1)	0 (0)	–	4
*Aedes geniculatus*	0 (0)	–	0 (0)	–	1 (2.4)	0.01 (0–1)	1
*Aedes cantans* ***/*** *annulipes*	0 (0)	–	0 (0)	–	10 (24.4)	0.05 (0–2)	10
*Aedes detritus*	3 (0.4)	0.02 (0–1)	68 (94.4)	0.35 (0–9)	0 (0)	–	71
*Aedes flavescens*	26 (3.2)	0.14 (0–6)	0 (0)	–	0 (0)	–	26
*Aedes punctor*	0 (0)	–	0 (0)	–	3 (7.3)	0.02 (0–1)	3
*Aedes rusticus*	0 (0)	–	0 (0)	–	6 (14.6)	0.03 (0–1)	6
*Aedes* spp.	1 (0.1)	0.01 (0–1)	1 (1.4)	0.01 (0–1)	0 (0)	–	2
*Anopheles maculipennis* (*s.l.*)^a^	54 (6.7)	0.28 (0–29)	0 (0)	–	1 (2.4)	0.01 (0–1)	55
*Anopheles plumbeus*	0 (0)	–	1 (1.4)	0.01 (0–1)	4 (9.8)	0.02 (0–1)	5
*Coquillettidia richiardii*	498 (62.1)	2.59 (0–67)	1 (1.4)	0.01 (0–1)	12 (29.3)	0.06 (0–2)	511
*Culex modestus*	199 (24.8)	1.04 (0–23)	0 (0)	–	0 (0)	–	199
*Culex pipiens* ^b^	16 (2.0)	0.08 (0–4)	0 (0)	–	2 (4.9)	0.01 (0–1)	18
*Culiseta annulata*	2 (0.3)	0.01 (0–1)	0 (0)	–	0 (0)	–	2
Total	802	4.18 (0–89)	72	0.38 (0–9)	41	0.21 (0–6)	915

^a^Includes both *Anopheles atroparvus* (van Thiel, 1927) and *Anopheles messeae* (Falleroni, 1926) / *Anopheles daciae* (Linton, Nicolescu & Harbach, 2004)

^b^Includes *Culex pipiens* f. *pipiens* and two specimens not identified to ecoform

### Generalized linear mixed models

**Fig. 3:**
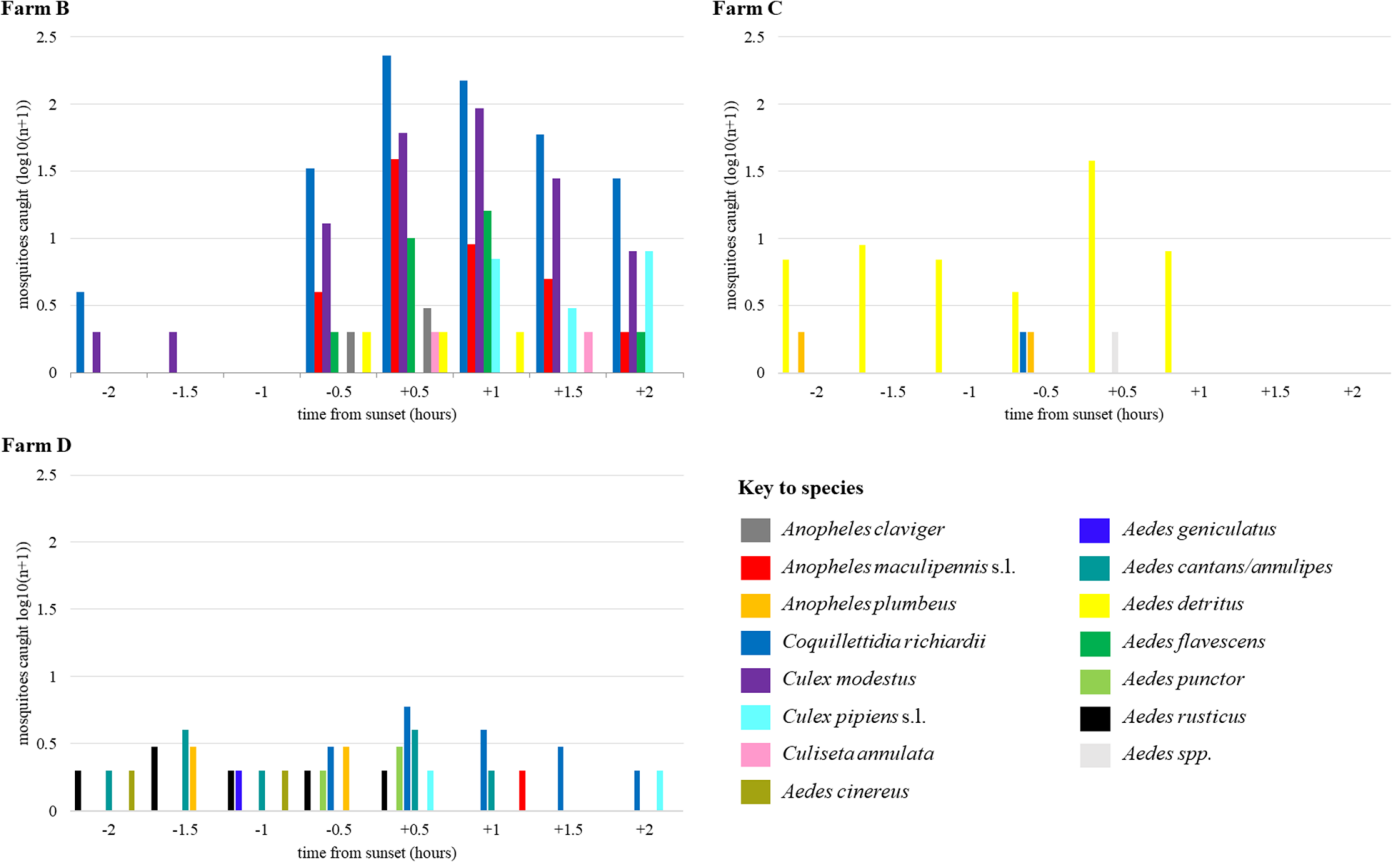
Mosquito biting activity relative to sunset. The log_10_ of the total mosquito species collected by human landing catch (all collectors) at farms B, C and D over the six visits to each farm. Farm A is excluded as no mosquitoes were collected there during the study

A negative binomial GLMM with logit link function was the best fit to the data and indicated that *site*, *time relative to sunset* and *wind speed* significantly influenced the total number of mosquitoes collected. Relative to the biting rate at site C, the total number of mosquitoes collected was significantly higher at site B (*P* ≤ 0.001), whilst there was a non-significant difference in collections between sites C and D (Additional file 14: Table S2). An increase of 1 m/s in wind speed would be expected to lead to a 58% decrease in the total biting rate (*P* ≤ 0.001), whilst the number of mosquitoes collected would be expected to decrease by 29% for every 30-min period further from sunset (*P* ≤ 0.001) (Additional file 14: Table S2). Temporal trends relative to sunset were the most pronounced for species collected at site B. For example, *Cq. richiardii* and *An. maculipennis* (*s.l.*) showed peak biting activity in the collection period shortly after sunset, *Cx. modestus* and *Aedes flavescens* (Müller, 1764) showed peak activity one hour after sunset, whilst *Cx. pipiens* f. *pipiens* was collected only after sunset, at sites B and D (Fig. 3).

Only *Cq. richiardii* and *Cx. modestus* were collected in sufficient number (*n* > 100) to permit individual analysis of abundance. As 495/511 (96.9%) of *Cq. richiardii* were collected from site B, the remaining specimens were excluded from the analysis and *site* omitted as a factor from the model. The best-fit negative binomial model for *Cq. richiardii* indicated that *time relative to sunset* and *wind speed* significantly influenced the biting activity of this species, predicting that a 1 m/s increase in the wind speed would lead to an estimated 41% decrease in biting rate (*P* ≤ 0.001) and every 30-min movement away from sunset would be expected to lead to an 18% decrease in the biting rate (*P* ≤ 0.001) (Additional file 14: Table S3). *Culex modestus* was only collected from site B and thus *site* was also omitted from this model. The best-fit negative binomial model indicated that only *time relative to sunset* was a significant predictor of the biting rate for *Cx. modestus*; for every 30-min movement away from sunset a 21% decrease in the biting rate would be expected (*P* ≤ 0.001) (Additional file 14: Table S4).

### Molecular species delineation and blood meal analysis

Species delineation by molecular methods of the 18 specimens identified morphologically as *Cx. pipiens/Cx. torrentium* indicated that only *Cx. pipiens* was present. Of these, 16/18 were identified as *Cx. pipiens* f. *pipiens*, whilst repeated attempts to identify the remaining two specimens proved unsuccessful. Of the 55 *An. maculipennis* (*s.l.*) collected, 50 were identified as *An. atroparvus* and five as *An. daciae/An. messeae*; the latter were collected only from site B and presented identical query results in BLAST searches, precluding their separation. Overall, therefore, sites B and D yielded nine species/species groups and site C five species.

Nineteen specimens comprising six species/species groups were found to contain blood in their abdomen (Table 3). Blood-feeding hosts were successfully identified in 12/19 specimens (63%). Ten blood meals were identified as being of human origin, with two blood meals, one from each of *An. atroparvus* and *Cq. richiardii*, identified as having originated from a cow (*Bos taurus* L.).

**Table 3:** Results of blood-meal analysis of mosquitoes collected on humans. Blood meals identified from engorged specimens collected by human landing catch at farms B, C and D

Species	Total blood-fed	Total (% positive for blood-meal host)	Blood-meal hosts (*n*)
*Aedes cantans*/*annulipes*	1	1 (100)	Human, *Homo sapiens* (1)
*Aedes detritus*	9	6 (67)	Human, *Homo sapiens* (6)
*Aedes flavescens*	2	0 (0)	na
*Anopheles atroparvus*	3	1 (33)	Cow, *Bos taurus* (1)
*Coquillettidia richiardii*	3	3 (100)	Human, *Homo sapiens* (2); Cow, *Bos taurus* (1)
*Culex modestus*	1	1 (100)	Human, *Homo sapiens* (1)
Total	19	12 (63)	

## Discussion

Quantifying mosquito biting rates on epidemiologically relevant hosts is a challenging undertaking, but is essential in order to understand the impact of nuisance-biting and the risk of mosquito-borne pathogen transmission. This study aimed to determine human-biting rates of native mosquitoes associated with four sites in southern England during the summertime evening peak of mosquito activity. Human-biting activity was identified on three of the four sites studied, despite the presence of alternative livestock and wildlife hosts in the area. Mosquito species assemblages differed between sites, but at least 15 species of seven genera were identified, including several proven or potential vectors of medically-important pathogens. Average biting rates differed significantly between farms: no human-biting was reported from site A in Oxfordshire, whilst biting rates at site B in Kent reached 89 mosquitoes in 25 min, or an extrapolated 214 bites per hour.

Biting rates at site B match those recorded at a site in Sandwich, Kent, some 40 miles south in 1981 [52] and match or exceed those reported from a wetland area in the Camargue, France, where West Nile virus (WNV) transmission has been reported [53]. The species composition of the biting population was significantly different to the earlier UK report, with the current study including *Cx. modestus* as the second most numerous species collected at the site. Feeding on both birds and mammals, *Cx. modestus* is an important bridge vector of WNV in mainland Europe [53–55]. *Culex modestus* is thought to have shown recent expansion in its distribution [56, 57] and has been targeted in WNV surveillance activities in the UK [30]. Although its establishment in the UK is considered a fairly recent event [58], this species has now been identified in several locations across the south of England [59]. To our knowledge, this study is the first to document the human-biting activity of *Cx. modestus* in the UK since an isolated report from the Portsmouth area in the 1940s [60].

Interestingly, the high abundance of human-biting mosquitoes identified at site B contrasts with parallel collections identifying only limited evidence of human feeding by mosquitoes at the same site [61] and an intensive study of blood-feeding behaviour conducted there in the subsequent year (2014), in which no human blood meals were identified [41]. The likely reasons for this are twofold. First, the mosquito behaviour post-feeding varies by species, with some being attracted to artificial resting traps [41], whilst others rest at other locations such as vegetation. This resulted in several of the major human-biting species collected in this study, such as *Cx. modestus*, being underrepresented in the resting collections, and others, such as *Anopheles maculipennis* (*s.l.*) and *Culiseta annulata* (Schrank, 1776), appearing under-represented in the human landing catch dataset. Secondly, the availability of humans to mosquitoes at their peak crepuscular biting times is normally relatively limited at the sites and, therefore, humans may not serve as a major blood meal source at these locations. This suggests that humans, although not necessarily a primary blood-feeding host, are readily fed upon when available by a range of mosquito species. Among these is the Pipiens ecoform of *Cx. pipiens*, collected from two sites in this study and which, despite being considered mainly ornithophilic, has also been reported to feed on humans and other mammals elsewhere [24]. Here, no blood-fed specimens were collected and therefore human feeding could not be confirmed by blood meal analysis. Nevertheless, collection by human landing catch indicates that this species may bite humans opportunistically. Taken together with experimental evidence for laboratory competence of mainland European *Cx. pipiens* f. *pipiens* for several arboviruses [62, 63], this species could therefore be considered a potential bridge, anthroponotic, and enzootic arbovirus vector in the UK.

As the aim of this study was to identify peak biting rates in ‘ideal’ flight conditions for mosquitoes, the range of meteorological data collected, and thus conclusions about the impact of each on biting activity, is limited. Nonetheless, wind speed was shown to be an important determinant of the total biting pressure experienced overall, and of the landing rates on humans of *Cq. richiardii* and *Cx. modestus* at site B. Although this result corresponds with existing findings demonstrating the importance of wind speed in influencing mosquito flight (e.g. [64, 65]), the requirement to use data from a station located 20 km away means this result should be viewed cautiously. Indeed, for future studies, we would advocate the assessment of meteorological variables at an even finer scale than attempted in this study, for example by collecting wind speed data from each sampling point, to capture within-site variation. Such fine-scale variation is an important factor to consider in rural environments as vegetation, in addition to artificial structures, can influence the meteorological conditions experienced within a small area [64].

The collections in this study targeted only the evening crepuscular biting period, in accordance with existing literature on the biting activity of UK mosquitoes [19] and the results of preliminary experiments. Peak mosquito biting occurred close to or shortly after sunset (Fig. 3), although this trend was more pronounced for certain species than others. Interestingly, *Aedes detritus* (Haliday, 1833) appeared to bite both before, during, and shortly after sunset, indicating that human exposure to biting by this arbovirus vector [7, 8] may begin earlier and persist for a longer period of time than with the other collected species. Studies conducted over a 24-h period (e.g. [53]) would be useful to provide a more complete picture of the mosquito biting risk posed to humans during other times of the day and night. For example, at least one of the sites (B) offers overnight stays to visitors and thus exposure to biting may occur beyond the evening period studied here. For example, species such as *Aedes rusticus* (Rossi, 1790) have been observed to bite during daylight hours in the UK (Nicholas Johnson personal communication). Further afield, 14-h landing catches conducted in the Ivory Coast to study the (normally daytime) biting activity of *Aedes aegypti* (L., 1762) found that biting by this species occurred throughout the night, with peak activity observed close to midnight [66].

One factor that was not considered in this study was the larval source of the species collected. This could be important as, particularly for smaller sites, the source of biting populations may be neighbouring habitats. Site D, for example, bordered an area of woodland larger than the farm itself. This area likely provided larval habitats for species such as *Ae. cinereus* (Meigen, 1818), *Ae. punctor* (Kirby, 1837) and *Ae. rusticus* which were collected exclusively from this site. Changes in species diversity and larval habitats could be determined by conducting human landing catches and larval sampling in a concentric radius, starting at the centre of each site and moving systematically into neighbouring habitat.

It is important from both a nuisance biting and pathogen transmission perspective to use a study design that most accurately reflects the true biting rate experienced by target hosts. The use of multiple volunteer collectors at four sampling points per site was designed to capture inter-individual differences in attractiveness to mosquito biting [21, 22], sampling efficiency [67] and within-site heterogeneity in exposure to biting [68]. No other European study has systematically assessed biting rate in this manner. In future, more representative data could be obtained by utilising moving, or roving, landing catches, which may better reflect the activity patterns of farm workers and visitors moving around on such sites. Human movement is an important but often overlooked component influencing biting and pathogen transmission risk [69]. Movement of a host may disrupt resting mosquitoes and provide an additional visual host stimulus causing mosquitoes to alight and feed. However, save for a few studies (e.g. [70–74]), moving catches remain a poorly explored method in studies of mosquitoes that bite humans.

## Conclusions

Potential and proven native mosquito vectors are responsible for considerable levels of human nuisance biting in some rural areas of southern England. *Coquillettidia richiardii*, *An. maculipennis* (*s.l.*) and *Cx. modestus* were the most common human-biting species, with peak hourly biting rates reaching 161, 70 and 55 bites, respectively. In practice, human exposure to peak mosquito biting activity in these areas may be limited to workers undertaking seasonal outdoor farming activities. Were a mosquito-borne pathogen outbreak to occur at the sampled sites, these activities may need to be altered to conclude prior to sunset and workers advised to implement bite prevention measures. Mosquito species assemblages biting humans, livestock and wildlife hosts suggest that rural sites such as farms could be areas where both enzootic and zoonotic pathogen transmission could occur. Therefore, such sites should be the subject of greater attention for future field studies of biting behaviour and risk analyses for pathogen transmission.

## Additional files

Additional file 1: **Table S1.** Further information on each farm used in this study. (PDF) (PDF 334 kb)

Additional file 2: **Figure S1.** Photosphere file, site B, sampling point 1. (JPG) (JPEG 2149 kb)

Additional file 3: **Figure S2.** Photosphere file, site B, sampling point 2. (JPG) (JPEG 1922 kb)

Additional file: **Figure S3.** Photosphere file, site B, sampling point 3. (JPG) (JPEG 1426 kb)

Additional file 5: **Figure S4.** Photosphere file, site B, sampling point 4. (JPG) (JPEG 1302 kb)

Additional file 6: **Figure S5.** Photosphere file, site C, sampling point 1. (JPG) (JPEG 1624 kb)

Additional file 7: **Figure S6.** Photosphere file, site C, sampling point 2. (JPG) (JPEG 1066 kb)

Additional file 8: **Figure S7.** Photosphere file, site C, sampling point 3. (JPG) (JPEG 2475 kb)

Additional file 9: **Figure S8.** Photosphere file, site C, sampling point 4. (JPG) (JPEG 1454 kb)

Additional file 10: **Figure S9.** Photosphere file, site D, sampling point 1. (JPG) (JPEG 1852 kb)

Additional file 11: **Figure S10.** Photosphere file, site D, sampling point 2. (JPG) (JPEG 2606 kb)

Additional file 12: **Figure S11.** Photosphere file, site D, sampling point 3. (JPG) (JPEG 1618 kb)

Additional file 13: **Figure S12.** Photosphere file, site D, sampling point 4. (JPG) (JPEG 2726 kb)

Additional file 14: **Table S2.** Regression coefficients, with Wald 95% confidence intervals and standard errors, for fixed effects of the best-fit negative binomial model used to describe total biting pressure. *** *P* ≤ 0.001. **Table S3.** Regression coefficients, with Wald 95% confidence intervals and standard errors, for fixed effects of the best-fit negative binomial model used to describe the biting activity of *Coquillettidia richiardii* (Ficalbi, 1889). *** *P* ≤ 0.001. **Table S4.** Regression coefficients, with Wald 95% confidence intervals and standard error, for fixed effects of the best-fit negative binomial model used to describe the biting activity of *Culex modestus* (Ficalbi 1889). *** *P* ≤ 0.001, * *P* ≤ 0.05. (PDF) (PDF 450 kb)

Additional file 15: **Table S5.** Data used in this study. (XLSX) (XLSX 44 kb)

## Abbreviations

AIC: Akaike information criterion
GLMM: generalized linear mixed model
PCR: polymerase chain reaction
WNV: West Nile virus
