# Supplementary material for: How often do mosquitoes bite humans in southern England? A standardised summer trial at four sites reveals spatial, temporal and site-related variation in biting rates
Source: Parasit Vectors. 2017 Sep 15;10:420. doi: 10.1186/s13071-017-2360-9 (PMC5602952; doi:10.1186/s13071-017-2360-9)
Supplement: Supplementary file 1 — Table S1. Further information on each farm used in this study. (PDF) (PDF 334 kb) [file 13071_2017_2360_MOESM1_ESM.pdf]

Additional file 1: Table S1

Further information on each farm used in this study.

Farm identifier	County	Co-ordinates	General description
A	Oxfordshire	Lat: 51.715807N Long: 1.380813W	Mixed inland livestock farm (cattle ~90, sheep ~1400 and horses ~8) close to other agricultural holdings. Close to the Thames and therefore is liable to winter and spring flooding.
B	Kent	Lat: 51.377445N Long: 0.784060E	Coastal grazing marsh maintaining cattle (750 site-wide) primary livestock species but sheep (~100) also maintained. Visitors present year-round due to popularity with bird watchers but main site closed to visitors at sunset.
C	Hampshire	Lat: 50.828166N Long: 0.962151W	Coastal dairy and arable farm maintaining ~120 head cattle. Many visitors to a tea room run by the farm on site.
D	Surrey	Lat: 51.322255N Long: 0.637692W	Smallholding close to ancient woodland and HMP Prison, maintaining ~50 head cattle.
