# Supplementary figures and images for: How often do mosquitoes bite humans in southern England? A standardised summer trial at four sites reveals spatial, temporal and site-related variation in biting rates
Source: Parasit Vectors. 2017 Sep 15;10:420. doi: 10.1186/s13071-017-2360-9 (PMC5602952; doi:10.1186/s13071-017-2360-9)

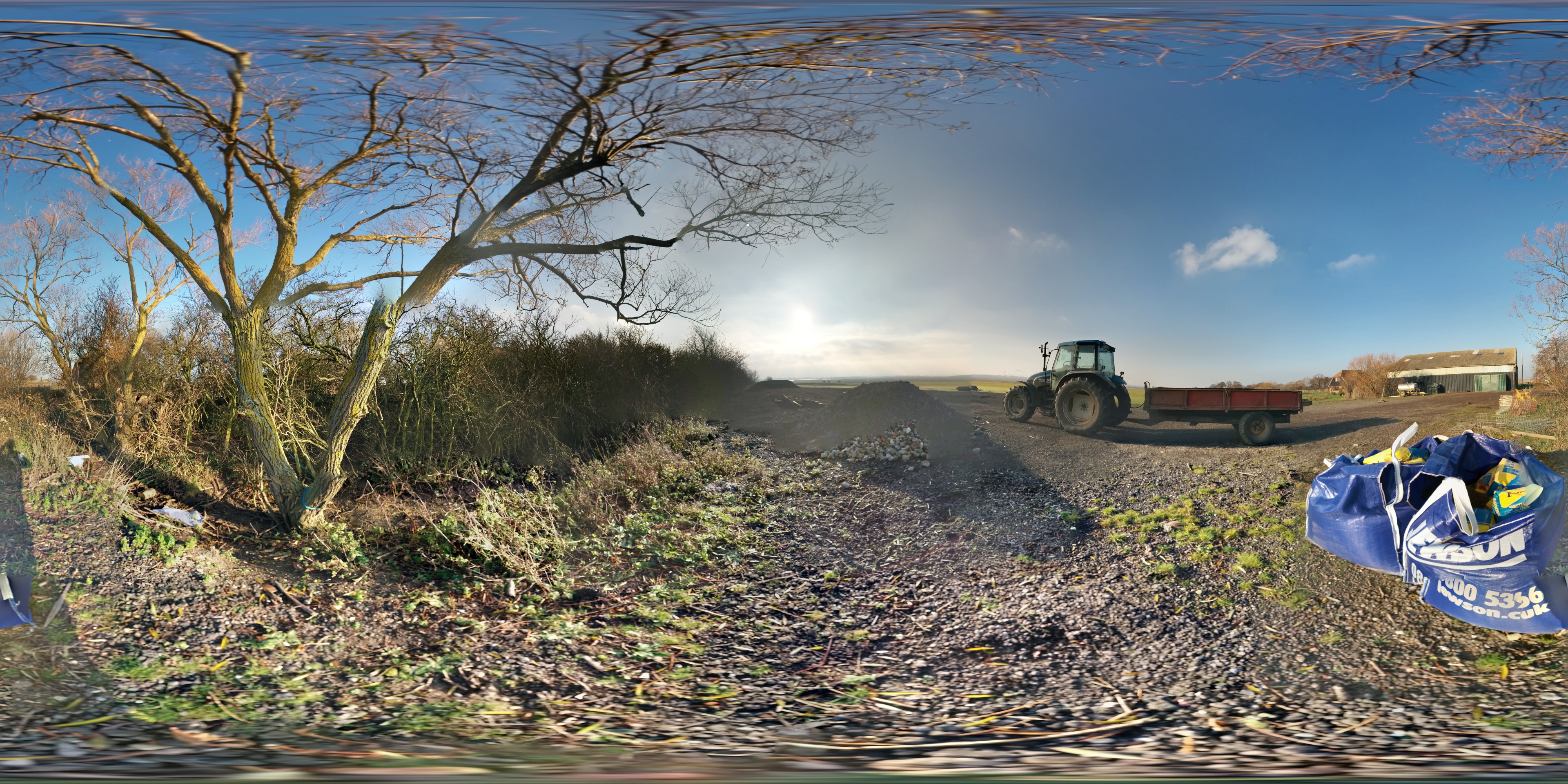

Supplement: Supplementary file 2 — Figure S1. Photosphere file, site B, sampling point 1. (JPG) (JPEG 2149 kb) [file 13071_2017_2360_MOESM2_ESM.jpg]

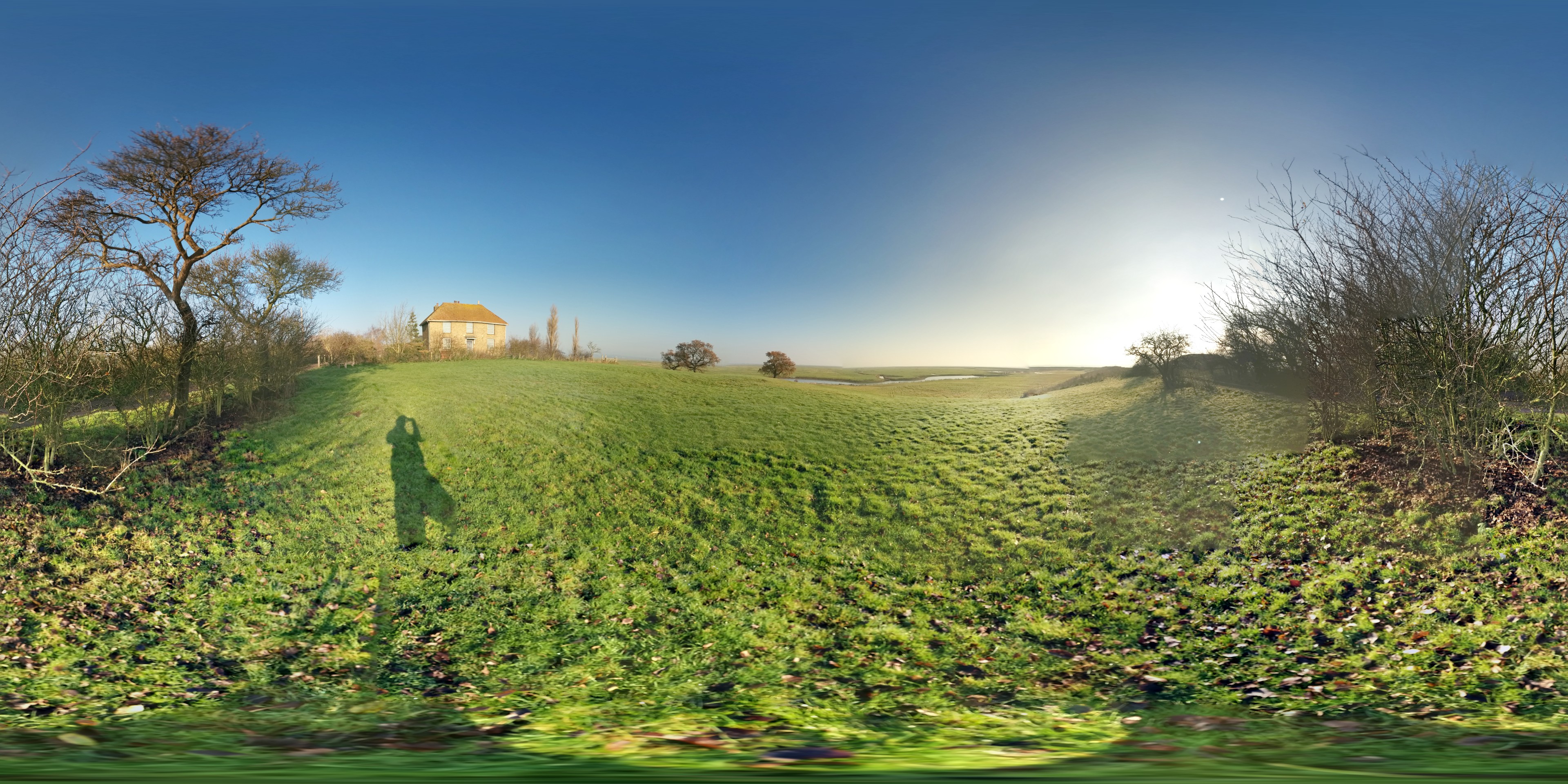

Supplement: Supplementary file 3 — Figure S2. Photosphere file, site B, sampling point 2. (JPG) (JPEG 1922 kb) [file 13071_2017_2360_MOESM3_ESM.jpg]

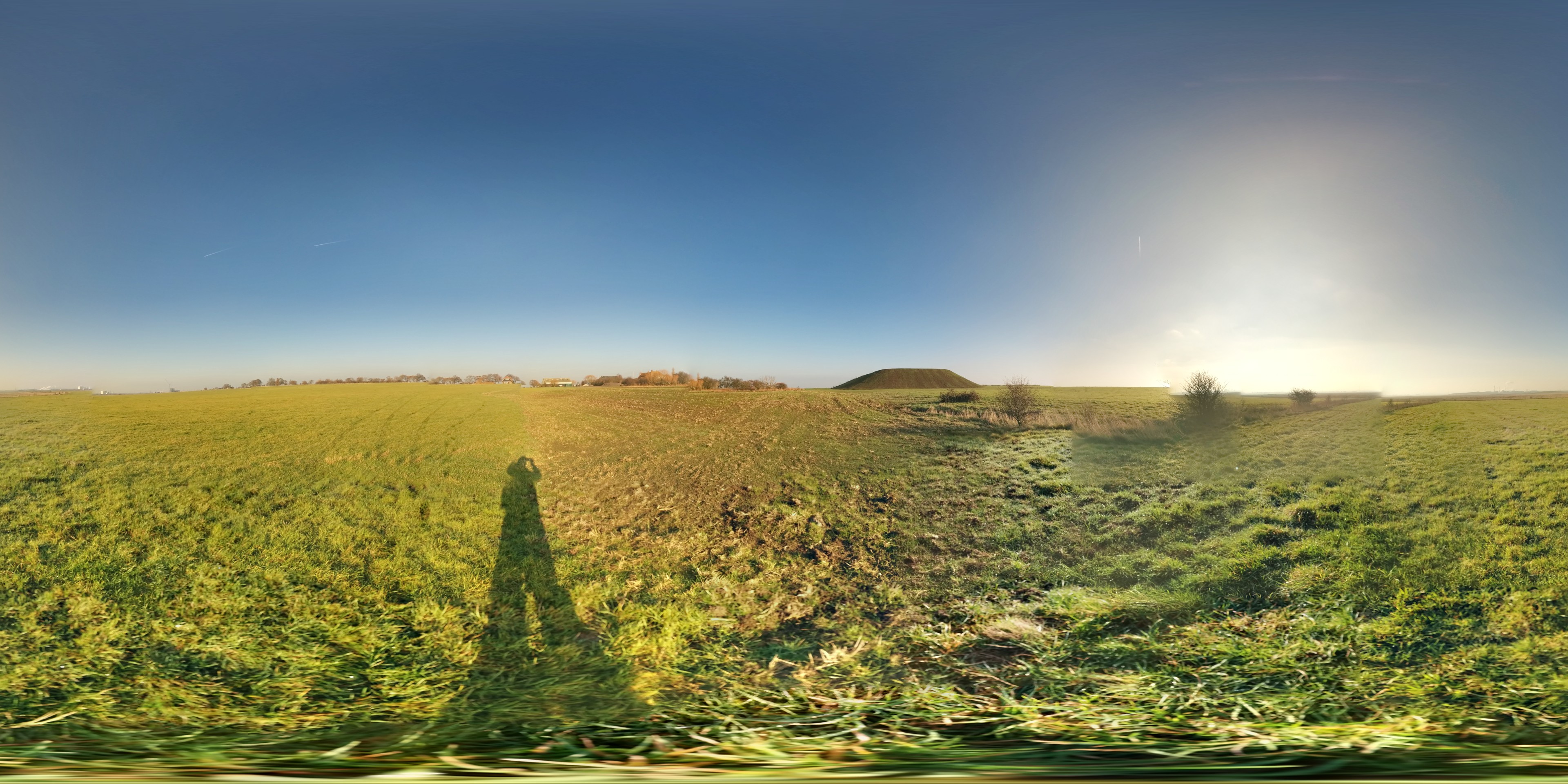

Supplement: Supplementary file 4 — Figure S3. Photosphere file, site B, sampling point 3. (JPG) (JPEG 1426 kb) [file 13071_2017_2360_MOESM4_ESM.jpg]

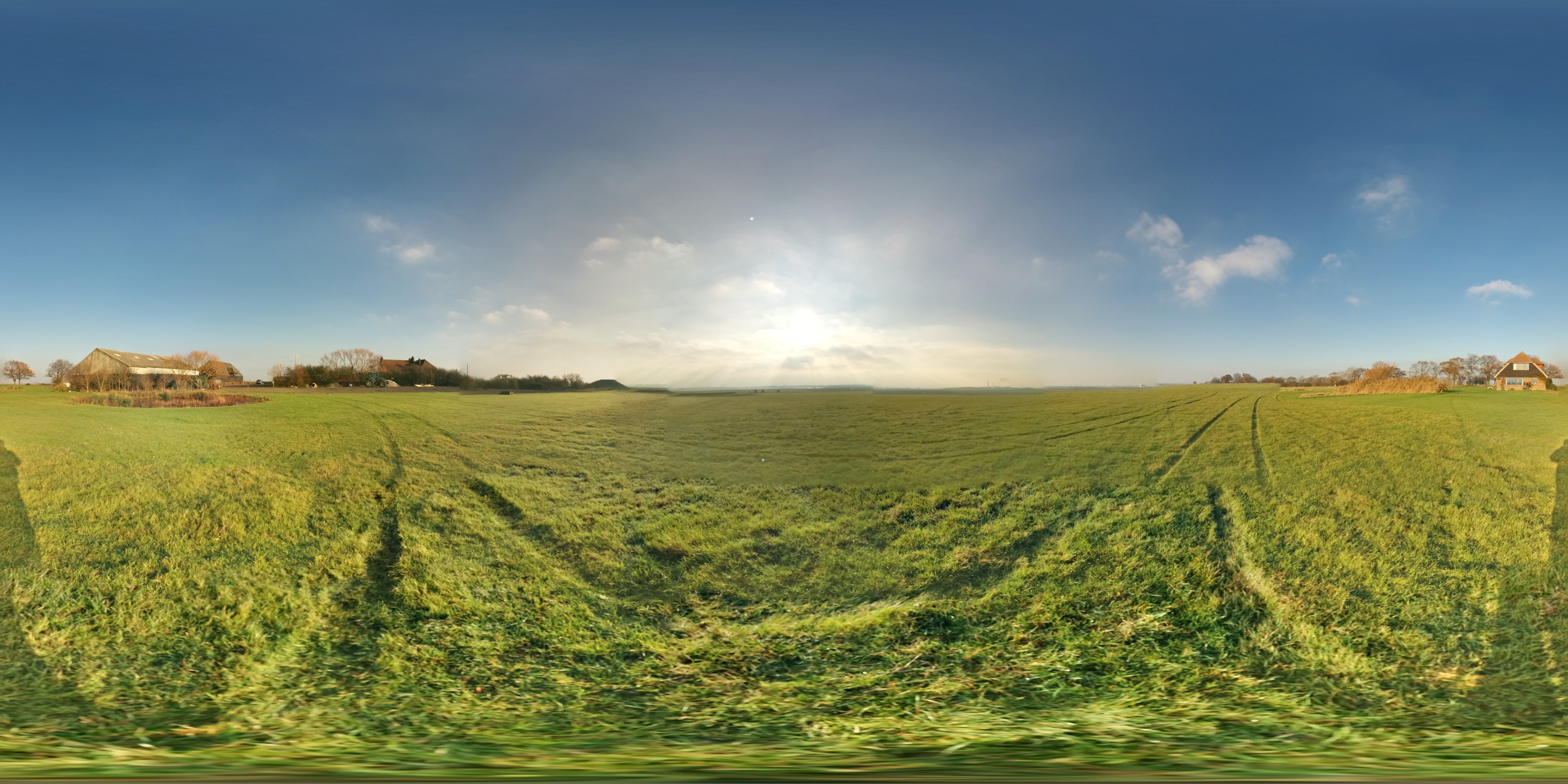

Supplement: Supplementary file 5 — Figure S4. Photosphere file, site B, sampling point 4. (JPG) (JPEG 1302 kb) [file 13071_2017_2360_MOESM5_ESM.jpg]

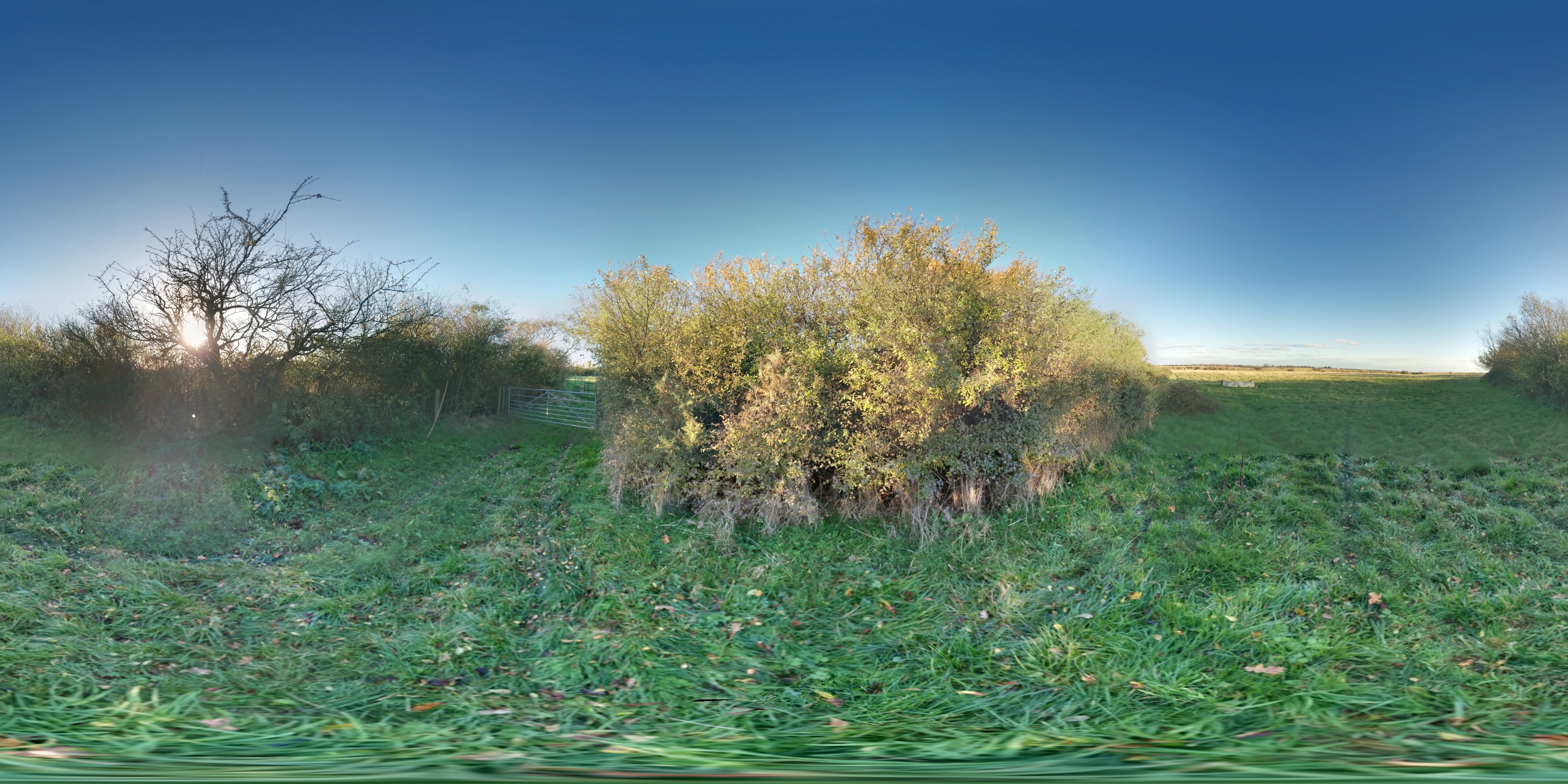

Supplement: Supplementary file 6 — Figure S5. Photosphere file, site C, sampling point 1. (JPG) (JPEG 1624 kb) [file 13071_2017_2360_MOESM6_ESM.jpg]

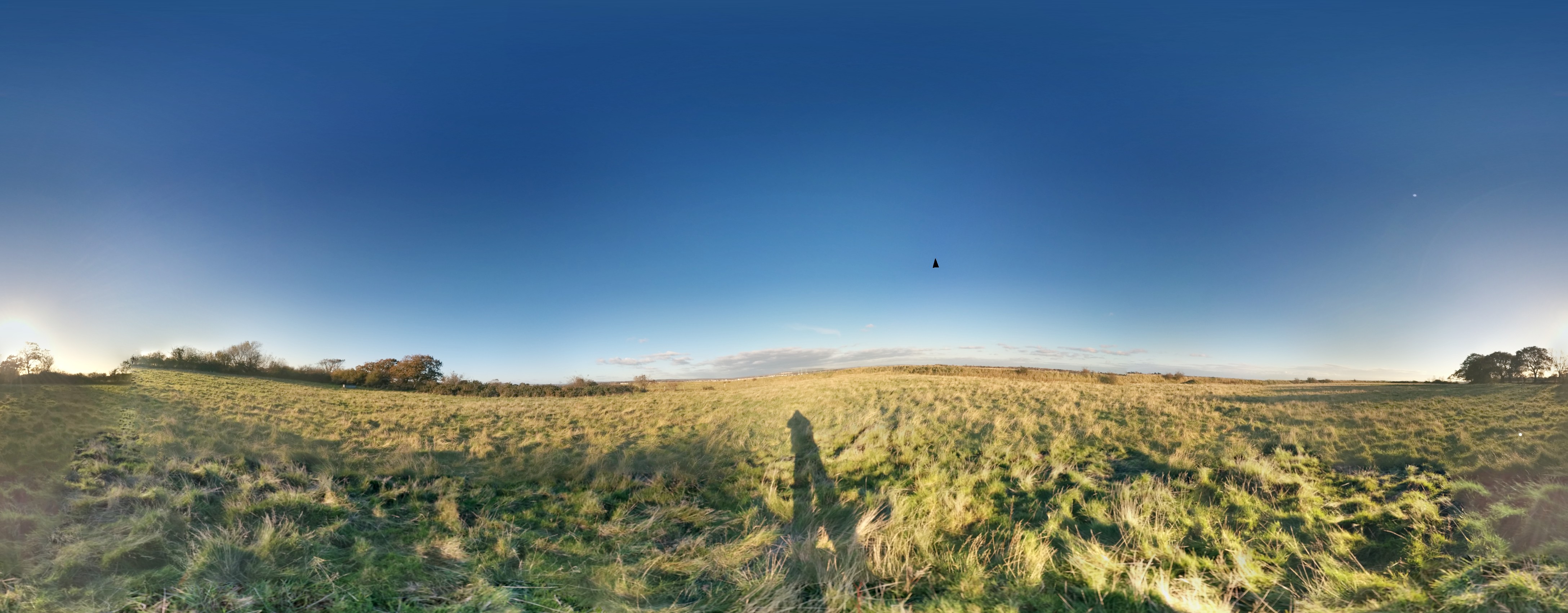

Supplement: Supplementary file 7 — Figure S6. Photosphere file, site C, sampling point 2. (JPG) (JPEG 1066 kb) [file 13071_2017_2360_MOESM7_ESM.jpg]

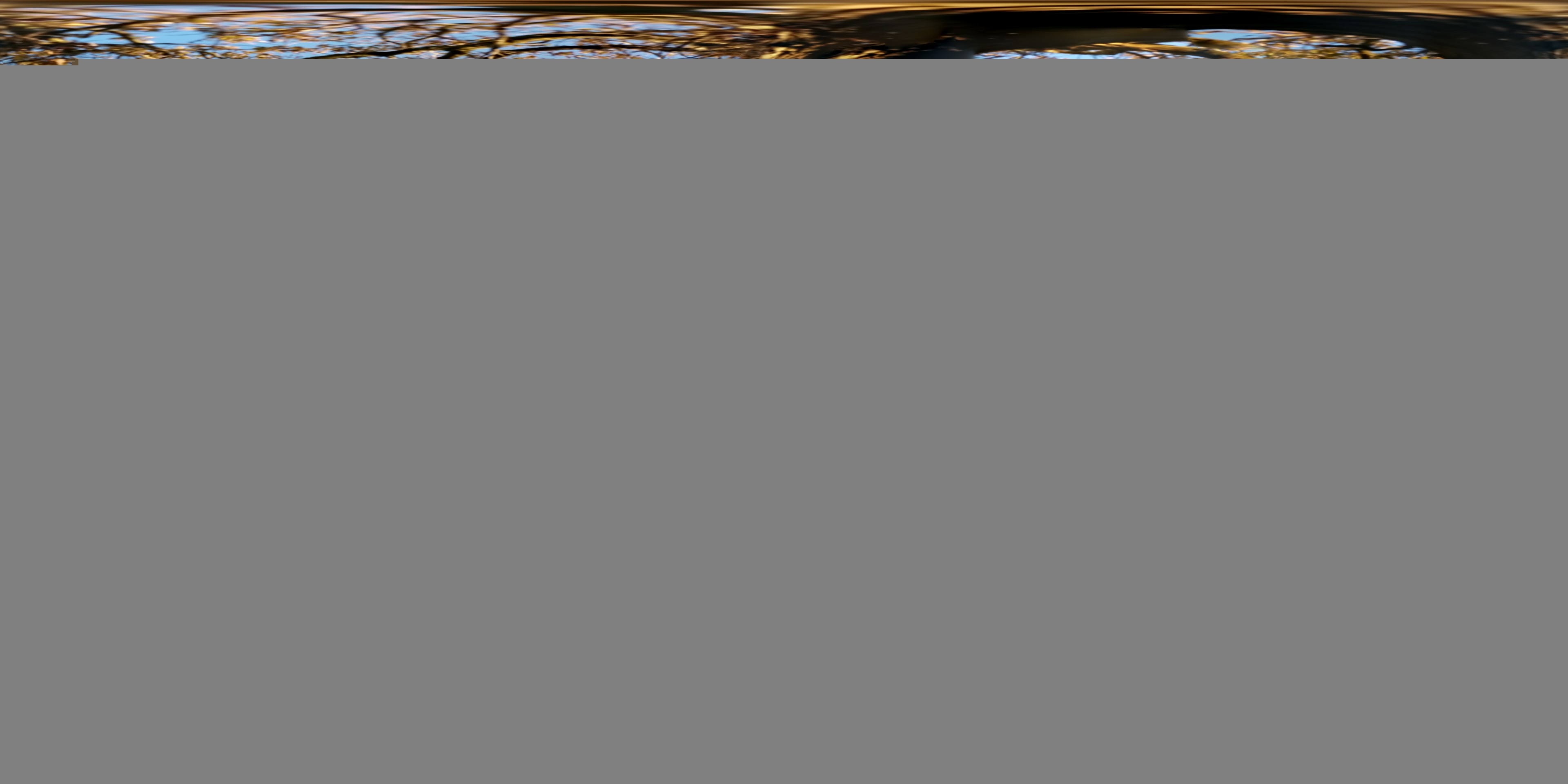

Supplement: Supplementary file 8 — Figure S7. Photosphere file, site C, sampling point 3. (JPG) (JPEG 2475 kb) [file 13071_2017_2360_MOESM8_ESM.jpg]

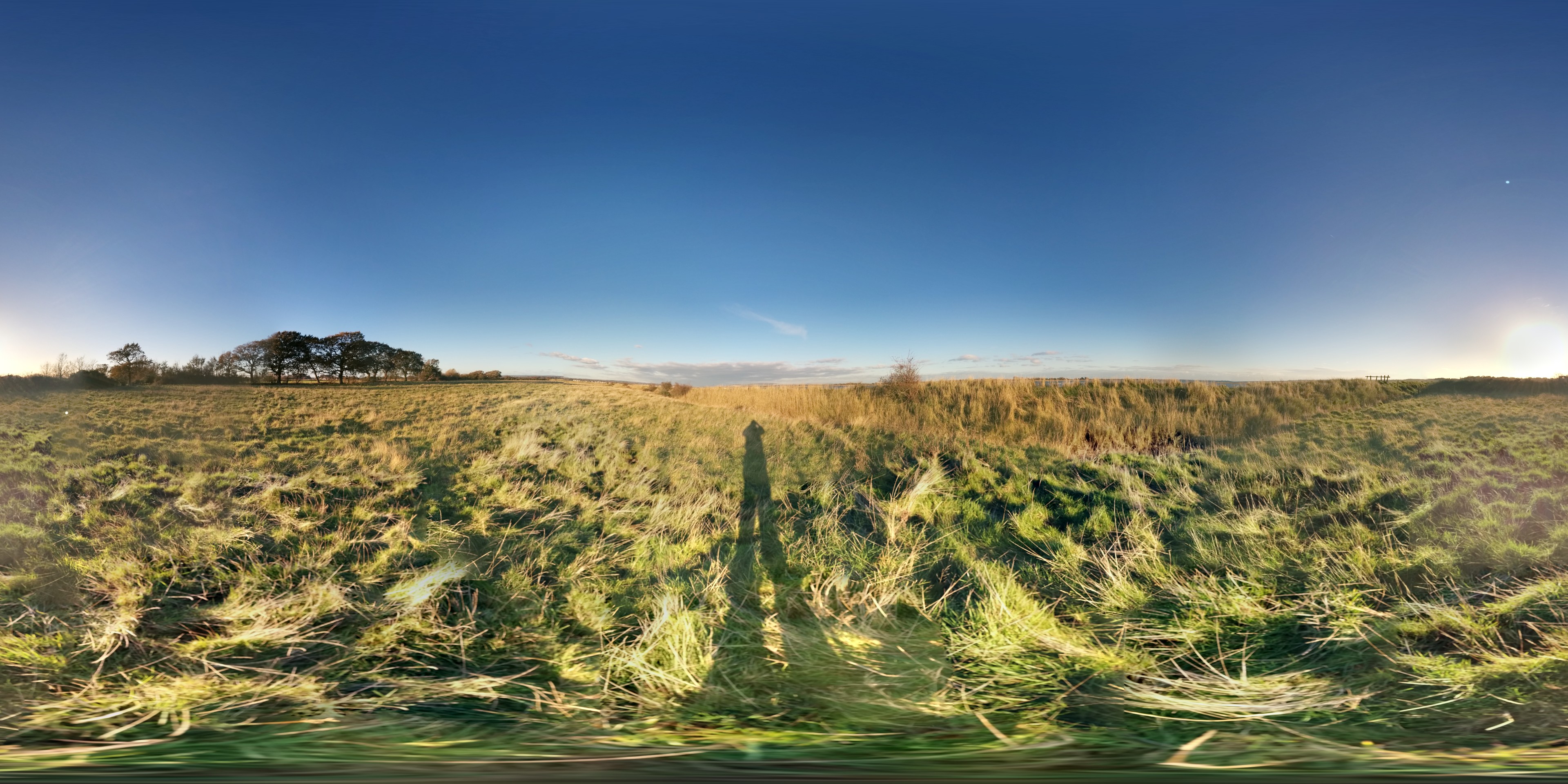

Supplement: Supplementary file 9 — Figure S8. Photosphere file, site C, sampling point 4. (JPG) (JPEG 1454 kb) [file 13071_2017_2360_MOESM9_ESM.jpg]

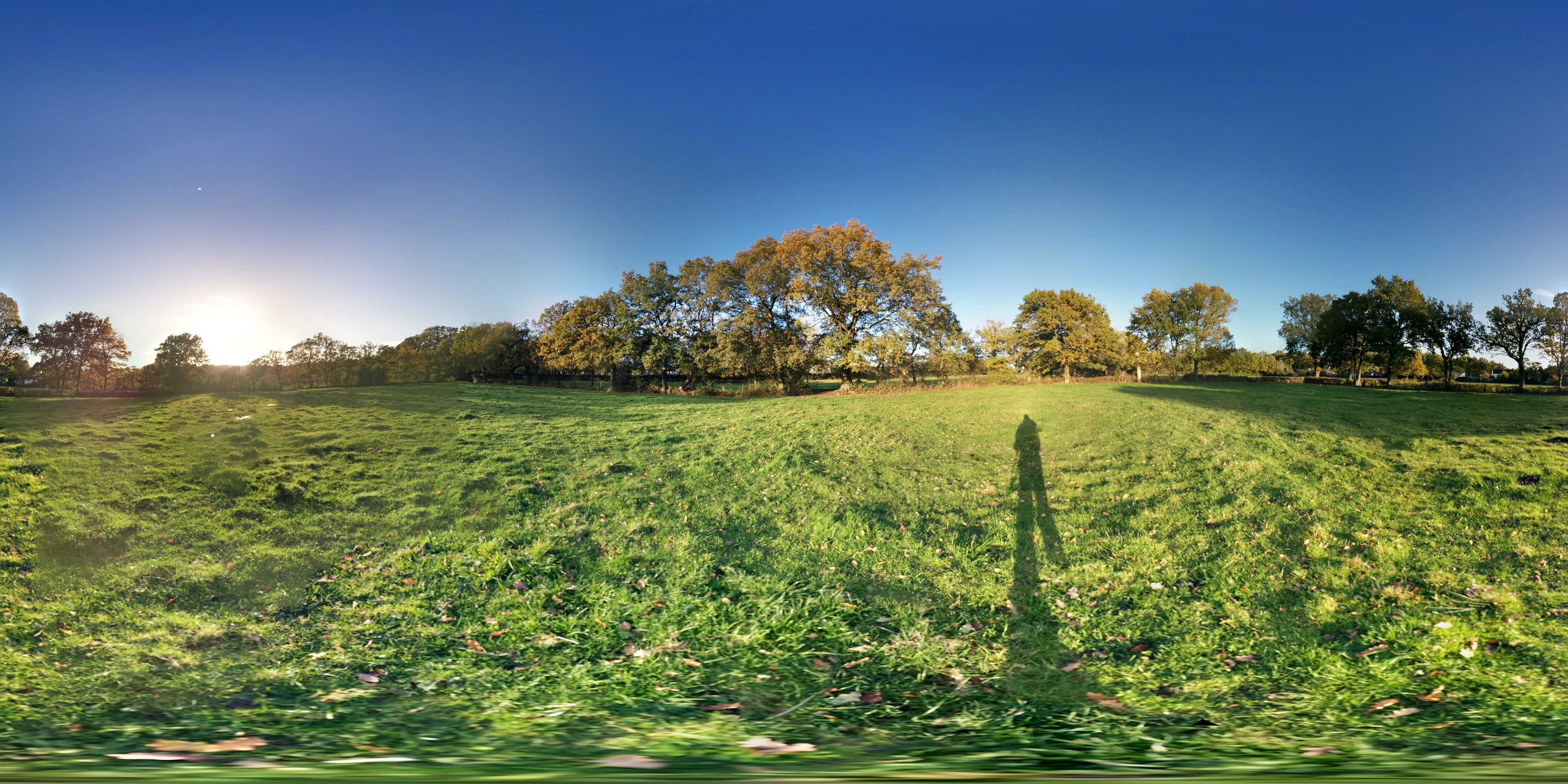

Supplement: Supplementary file 10 — Figure S9. Photosphere file, site D, sampling point 1. (JPG) (JPEG 1852 kb) [file 13071_2017_2360_MOESM10_ESM.jpg]

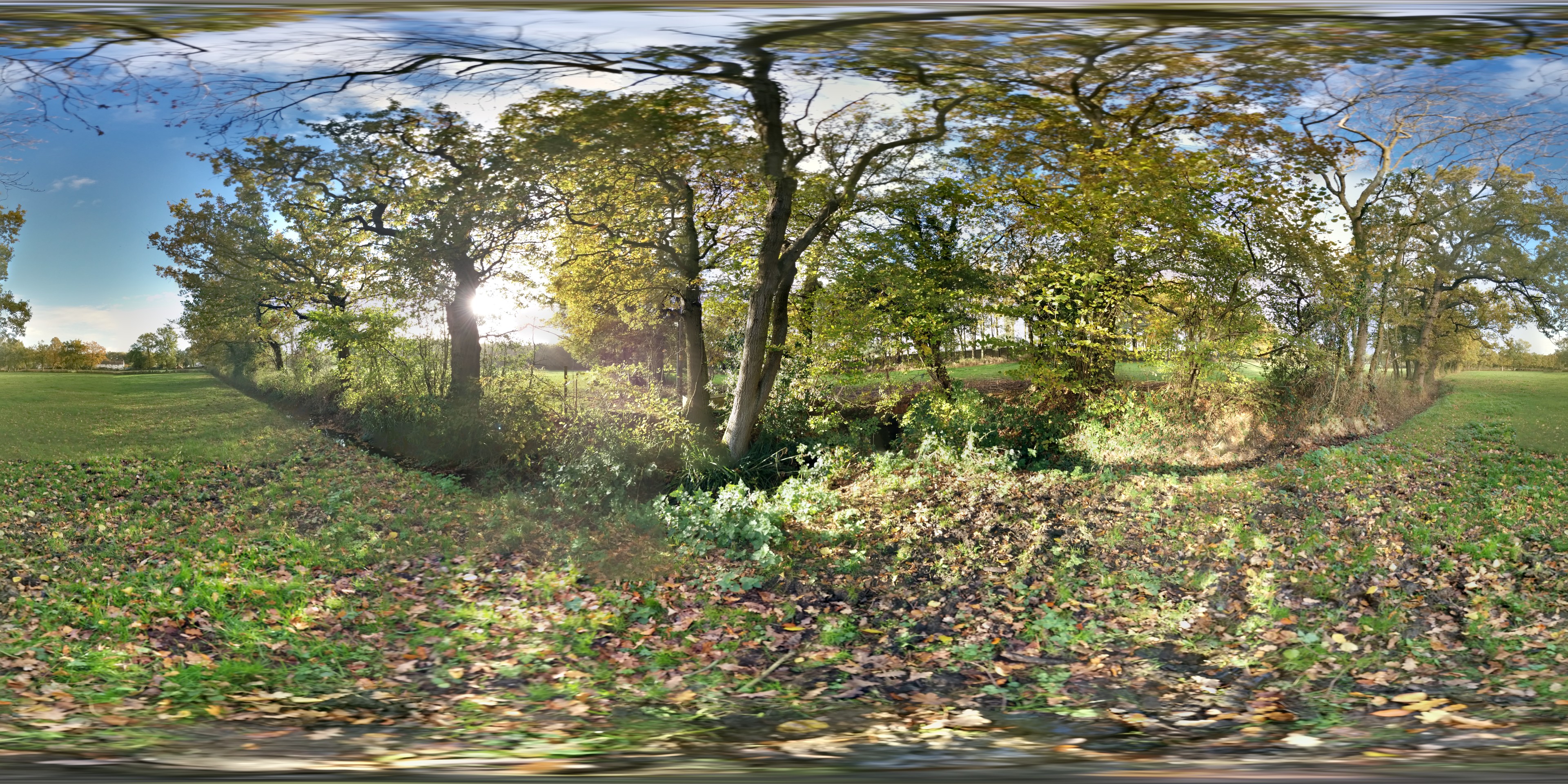

Supplement: Supplementary file 11 — Figure S10. Photosphere file, site D, sampling point 2. (JPG) (JPEG 2606 kb) [file 13071_2017_2360_MOESM11_ESM.jpg]

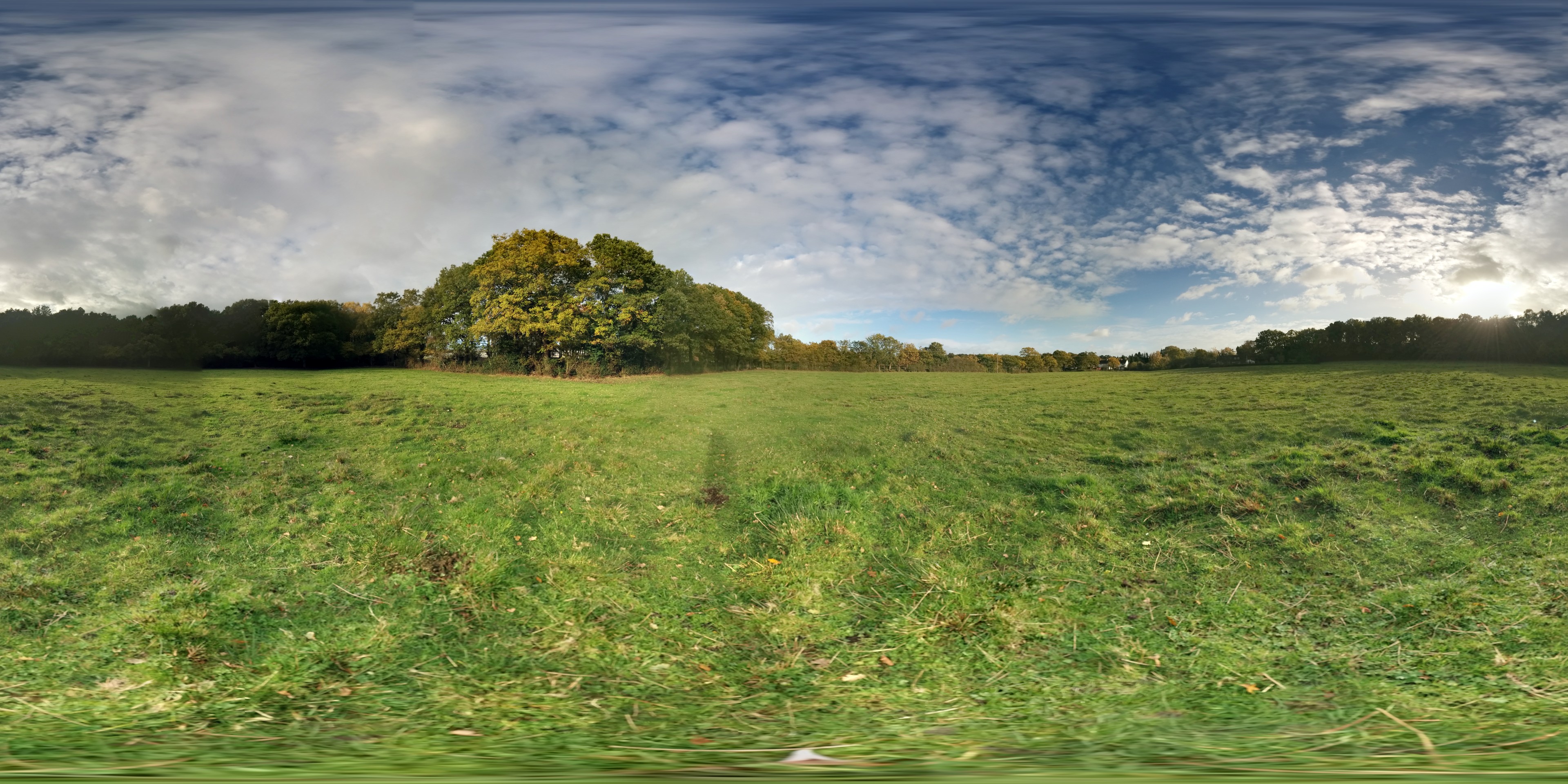

Supplement: Supplementary file 12 — Figure S11. Photosphere file, site D, sampling point 3. (JPG) (JPEG 1618 kb) [file 13071_2017_2360_MOESM12_ESM.jpg]

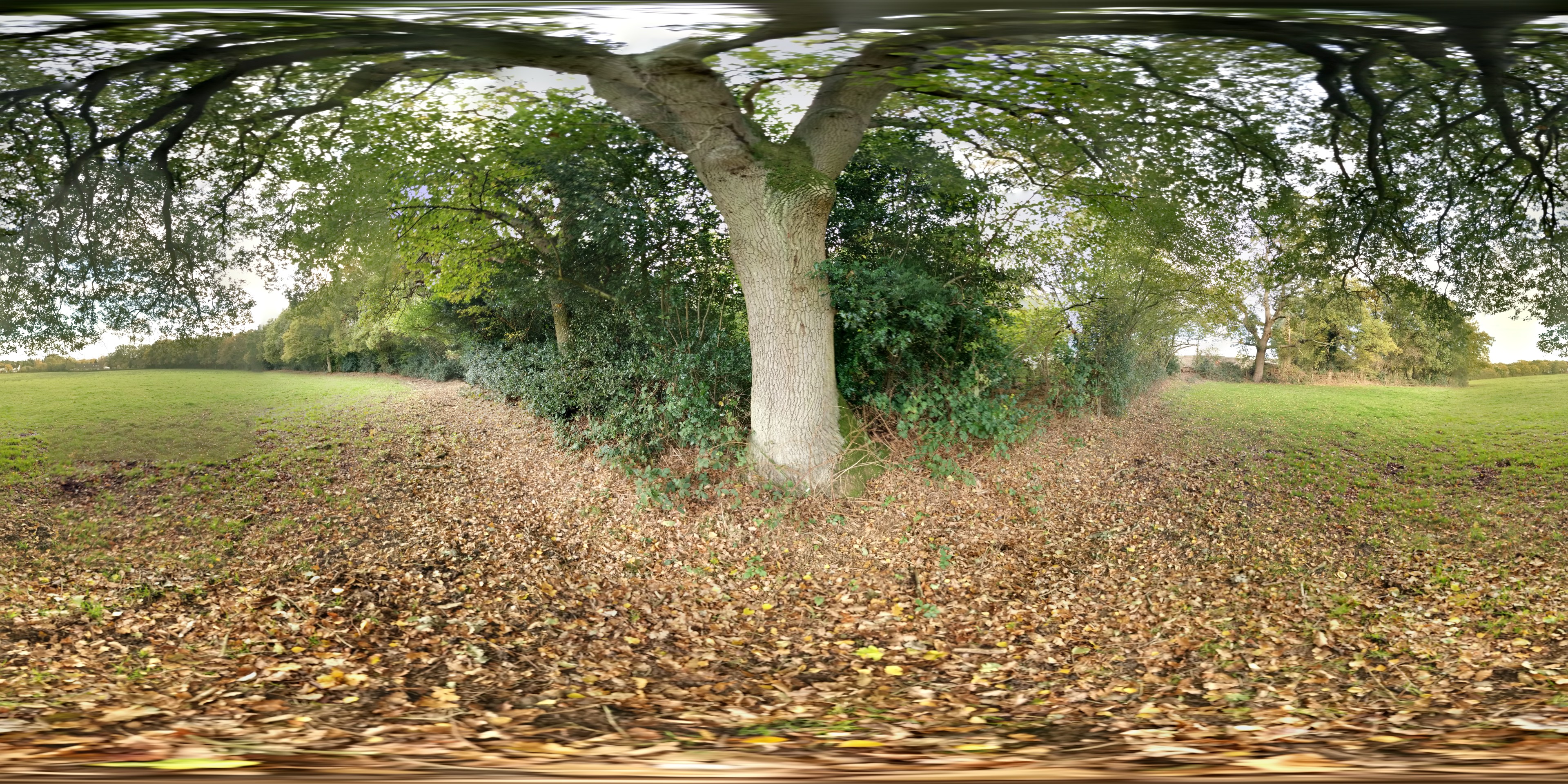

Supplement: Supplementary file 13 — Figure S12. Photosphere file, site D, sampling point 4. (JPG) (JPEG 2726 kb) [file 13071_2017_2360_MOESM13_ESM.jpg]
