# Supplementary material for: How often do mosquitoes bite humans in southern England? A standardised summer trial at four sites reveals spatial, temporal and site-related variation in biting rates
Source: Parasit Vectors. 2017 Sep 15;10:420. doi: 10.1186/s13071-017-2360-9 (PMC5602952; doi:10.1186/s13071-017-2360-9)
Supplement: Supplementary file 14 — Table S2. Regression coefficients, with Wald 95% confidence intervals and standard errors, for fixed effects of the best-fit negative binomial model used to describe total biting pressure. *** P ≤ 0.001. Table S3. Regression coefficients, with Wald 95% confidence intervals and standard errors, for fixed effects of the best-fit negative binomial model used to describe the biting activity of Coquillettidia richiardii (Ficalbi, 1889). *** P ≤ 0.001. Table S4. Regression coefficients, with Wald 95% confidence intervals and standard error, for fixed effects of the best-fit negative binomial model used to describe the biting activity of Culex modestus (Ficalbi 1889). *** P ≤ 0.001, * P ≤ 0.05. (PDF) (PDF 450 kb) [file 13071_2017_2360_MOESM14_ESM.pdf]

Additional file 14

Coefficients	Estimate (95% CI)	Standard error
(Intercept)	0.716 (-0.33; 1.76)	0.534
Farm C	-1.160 (-2.40; 0.08)	0.631
Farm B	2.870 (1.72; 4.02) ***	0.589
Time rel. to sunset	-1.236 (-1.65; -0.82) ***	0.211
Wind speed	-0.541 (-0.78; -0.30) ***	0.123

Table S2: Regression coefficients, with Wald 95% confidence intervals and standard errors, for fixed effects of the best-fit negative binomial model used to describe total biting pressure. *** $P \leq 0.001$.

Coefficients	Estimate (95% CI)	Standard error
(Intercept)	4.440 (2.99; 5.89) ***	0.741
Time from sunset	-1.741 (-2.46; -1.02) ***	0.368
Wind speed	-0.888 (-1.25; -0.52) ***	0.189

Table S3: Regression coefficients, with Wald 95% confidence intervals and standard errors, for fixed effects of the best-fit negative binomial model used to describe the biting activity of *Coquillettidia richiardii*. *** $P \leq 0.001$.

Coefficients	Estimate (95% CI)	Standard error
(Intercept)	1.688 (0.58; 2.79) *	0.564
Time from sunset	-1.526 (-2.39; -0.66) ***	0.441

Table S4: Regression coefficients, with Wald 95% confidence intervals and standard error, for fixed effects of the best-fit negative binomial model used to describe the biting activity of *Culex modestus*. *** $P \leq 0.001$, * $P \leq 0.05$.
